# Lower Extremity Injuries Due to Chainsaw During Four-Year Periods

**DOI:** 10.7759/cureus.44058

**Published:** 2023-08-24

**Authors:** Çağatay Gemci, Ahmet Imerci, Nevres Hurriyet Aydogan

**Affiliations:** 1 Department of Orthopaedics and Traumatology, Bursa Yuksek Ihtisas Training and Research Hospital, Bursa, TUR; 2 Department of Orthopaedics and Traumatology, Mugla Sitki Kocman University Hospital, Mugla, TUR

**Keywords:** ankle and foot, extensor tendon rupture, lower extremity trauma, traumatic injury, chainsaw

## Abstract

Background

Chainsaws cause injuries mostly on the upper extremities, then on the face and lower extremities. In the literature, there are many studies about hand and face injuries; however, articles about lower extremity injuries are limited. The aim of the study is to define injury patterns, treatments, and results of the cases that we have encountered in our hospital and to evaluate precautions after reviewing the literature.

Methods

Patients admitted to our hospital’s Emergency Department with chainsaw-related lower extremity injuries between 2016 and 2021 are evaluated. Patients’ demographic data, pathologies, treatments, length of stay in hospital, return to work time, and functional scores are calculated retrospectively.

Results

There were 39 male and two female patients, with a minimum follow-up of 12 months. Their mean age was 42.6 ± SD (16-62). Thirty-two patients (78.04%) had injuries on the left lower extremity, and nine patients (21.9%) had injuries on the right lower extremity. 93.75% (30/32) of the patients with left lower extremity injuries had the right hand as the dominant extremity. The most frequently observed injury pattern was extensor hallucis longus (EHL) tendon disruption, with a percentage of 58.5% (24/41). 29.2% (13/41) of the cases had bone pathologies present as well. Patients’ average AOFAS score was 97.4 ± 4.4 (74-100) at the end of one year. The average hospitalization length of stay was 2.95 ± 2.7 (0-15) days, and the time interval of return to work was 6.17 ± 1.4 (2-15) weeks, excluding one patient who had to change his workplace.

Conclusion

Saw-related injuries of the lower extremities are the injuries that can be seen mostly in male patients. Among the right dominant-handed patients, left foot dorsum injuries were the most common EHL tendon disruptions observed. We have seen that the reason for this is foot injuries remaining in the projection of the saw due to incorrect positioning of the foot. Protective gear and shoes must be used as precautions. More preventive measures could be taken while using chainsaws and similar tools, as they may cause serious injuries. Requirements for the use and sale of this tool should be introduced, and training should be given as it can be easily purchased by the public.

## Introduction

Chainsaws are common tools for professional woodworking, metalworking, carpentry, hobbies, and household use. Manufacturers offer these products with safety booklets, but it is not known whether users are using them properly or whether they have safety equipment. Injuries and loss of work associated with chainsaws have been reported in the literature in carpentry and woodworking. [1,2]. These devices can cause serious injuries to all parts of the body when used inappropriately by inexperienced or inadequately trained people or without safety equipment. The rotating chain of the saw can cause laceration of vessels, nerves, and tendon structures, but it is strong enough to go through injuries ranging from bone tissue destruction to amputation [3,4].

In the literature, it has been frequently described as maxillofacial and hand injuries, but injuries involving the lower extremity are rarely described [5,6]. Characteristically, chainsaw injuries are in the form of scorching, heat-induced burns, and maceration, as described in the literature [5]. In a recent study, it was shown that 26% of patients had injuries involving bone pathology in the lower extremity [7].

The preventable nature of chainsaw accidents and the impact of the lower extremity were instrumental in the decision to undertake a comprehensive investigation. These injuries could lead to the loss of fingers or extremities, the loss of jobs, or a significant number of workdays. Therefore, we aimed to examine the specific lower extremity injury characteristics and treatment modalities associated with chainsaw use among patients admitted to the emergency department of our hospital. We also reviewed the literature to better define methods of protection from these injuries today and in the future.

## Materials and methods

Between 2016 and 2021, 54 patients with lower extremity injuries related to chainsaw injuries were detected in the emergency service of our university training and research hospital. Thirteen patients had skin lacerations that would not require surgical intervention and were not included in the study. Forty-one patients with lower extremity injuries who underwent orthopedic intervention were included in the study. Ethics Committee approval has been obtained. Physical examinations, AP and lateral radiographs, and, if necessary, computed tomography images were evaluated when all patients were admitted to the emergency department. Age, gender, mechanism and area of injury, referral time after injury, type of treatment, and functional results were analyzed retrospectively in all patients. The months of admission, hospital length of stay, occupation, and return to work of the patients were recorded. Functional scores in all cases were evaluated by the American Orthopedic Foot and Ankle Society Score (AOFAS) at one year postoperatively. Since these injuries usually present as a fragmented, burn-like appearance caused by heat and chain, tetanus prophylaxis and intravenous antibiotics were applied in the emergency room as the first step of treatment. All cases were operated on within the first 12 hours. Extensive debridement and irrigation were applied to all patients during the operation. No large soft tissue defect requiring a flap was detected in any of the patients. No implant was used for fixation in injuries with periosteal stripping and incomplete fractures, and conservative treatment was chosen. Fixation with Kirschner wire was applied to all phalanx fractures. Tendon and soft tissue repairs were performed after debridement. Low molecular weight heparin (LMWH) was applied during immobilization in patients with fractures.

Statistical analysis

Parametric and non-parametric analyses were performed. Descriptive and frequency statistics were used to describe the population by mean and standard deviation. Statistical analysis was performed using IBM Corp. Released 2017. IBM SPSS Statistics for Windows, Version 25.0. Armonk, NY: IBM Corp. The statistical significance of risk factors was evaluated at level 0.05.

## Results

**Figure 1:**
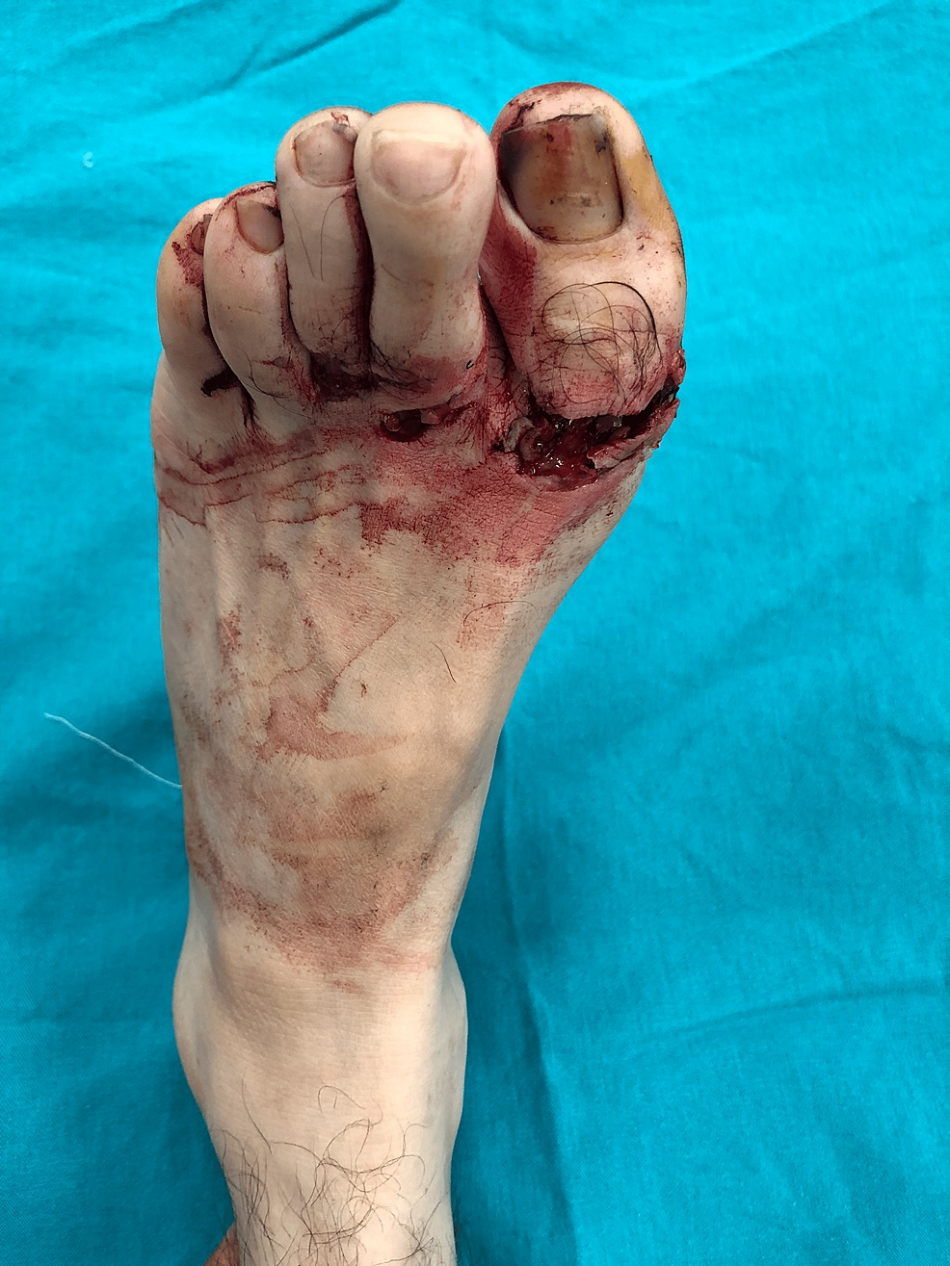
61-year-old male patient with left foot EHL disruption EHL: Extensor hallucis longus

The mean age of the patients with chainsaw injuries admitted to our hospital was 43.4 ± 12 (16-62). Two of the patients were female, and 39 were male (Table 1). While five of the patients were dominantly left-handed, 36 of them were right-handed. It was learned that all patients were injured while doing their own work at home. Injuries were frequently seen in January (13 cases) and November (11 cases). Autumn and winter months, February (three cases), September (two cases), and October (two cases), covered 75.6% of the total cases. None of the subjects stated that they did not wear protective shoes or boots at the time of the injury. Professionally, only four patients were engaged in forestry. Thirty-seven patients stated that they were injured while using the chainsaw for their own gardening. There were left lower extremity injuries in 32 patients (78,04%) and right lower extremity injuries in nine patients (21,16%). Of the 32 patients whose left lower extremity was injured, 30 had the right hand dominant; only two patients had left hand dominance (p = 0.012). Of nine patients whose right lower extremity was injured, six were right-hand dominant, and three were left-hand dominant (Table 2). Most of the injuries (26 cases, 63.4%) were in the dorsal region of the left foot. It was determined that the injury usually occurred because the left foot was in front of the chainsaw handle or in the projection position of the saw. All of the injuries occurred after contact with the chain. The most common injury was the extensor hallucis longus (EHL) tendon cut, which was seen in 24 cases (58.5%) (Figure 1). In 12 patients (29.2%), osseous pathologies were detected as incomplete or complete fractures. Eight patients (19.5%) had a tibialis anterior tendon injury. In seven patients (17%), the second and third finger extensor tendons were accompanied by other injuries (Table 3).

**Table 1:** Patient data of injury, treatment, and results AOFAS: American Orthopedic Foot and Ankle Society Score, EHL: Extensor hallucis longus, TAT: Tibialis anterior tendon, FHL: Flexor hallucis longus, TPT: Tibialis posterior tendon, MTP: Metatarsophalangeal, FDC: Flexor digitorum communis, EDC: Extensor digitorum communis

Patients' No.	Side	Age	Dominant hand	Injury	Treatment	AOFAS	Return to work (week)	Stay in hospital (day)
1	L	55	R	EHL injury	Primary repair	100	6	3
2	L	59	R	EHL injury	Primary repair	100	5	1
3	L	47	R	TAT injury	Primary repair	100	6	1
4	L	61	R	EHL injury + 1 MTP joint capsule injury + 1 Finger proximal phalanx incomplete fracture	Primary repair	98	6	3
5	L	25	R	EHL, 2 and 3 extensor tendon injury + 2 Finger proximal phalanx comminuted fracture	Primary repair + debridement + K-wire fixation	95	8	15
6	R	45	R	Partial quadriceps tendon injury	Primary repair	100	8	3
7	L	33	R	EHL injury and 2 extensor tendon injury + 1 Finger proximal phalanx incomplete fracture	Primary repair + debridement	100	6	3
8	R	42	R	1 Finger subtotal amputation	EHL, FHL, digital nerve repair, K-wire fixation	95	Changed work	12
9	L	16	R	EHL injury and partial TAT injury	Primary repair	100	7	3
10	L	61	R	EHL injury	Primary repair	100	6	3
11	L	50	R	EHL injury + 1 Metatarsal incomplete fracture	Primary repair	100	8	3
12	L	30	R	EHL injury	Primary repair	100	2	0
13	L	55	R	Superficial deltoid ligament injury+ Partial TAT injury	Primary repair	95	4	5
14	L	26	R	EHL injury + 1 Metatarsal incomplete fracture + digital nerve injury	Primary repair + K-wire fixation	98	6	3
15	L	36	R	EHL injury + TAT injury	Primary repair	96	7	3
16	R	62	L	EHL injury	Primary repair	100	6	2
17	L	46	R	TAT injury, EHL injury, EDC injury, medial cuneiform, navicular incomplete fracture, dorsalis. pedis arterial injury	Primary repair	94	12	4
18	L	50	R	EHL injury + 1 Finger proximal phalanx incomplete fracture	Primary repair + K-wire fixation	96	5	3
19	L	37	R	EHL + 2 EDC injury	Primary repair	100	6	3
20	R	54	R	MCL partial injury on the knee	Primary repair	100	2	1
21	L	44	L	1 MTP joint capsule injury	Primary repair	100	2	2
22	L	39	R	EHL and joint capsule injury	Primary repair	100	5	2
23	L	22	R	EHL injury + proximal phalanx incomplete fracture	Primary repair + K-wire fixation	98	6	3
24	R	43	R	Peroneal nerve injury on cruris level	Primary repair (Operated by Plastic and Reconstructive Surgeon)	93	10	3
25	L	42	R	Medial malleolus incomplete fracture	Debridement + cast	100	8	1
26	R	54	L	EHL, 2 and 3 EDC injury	Primary repair	100	6	2
27	L	47	R	1 and 3 Finger subtotal, 2 Finger total amputation	1.and 3. fingers K-wire fixation, extensor tendon repair	74	15	7
28	L	32	R	EHL and TAT injury	Primary repair	97	7	3
29	R	51	R	EHL partial injury	Primary repair	100	4	0
30	L	54	R	EHL injury	Primary repair	98	6	2
31	L	22	L	TAT injury	Primary repair	94	6	2
32	L	30	R	TPT injury	Primary repair	97	6	2
33	R	40	L	EHL injury	Primary repair	100	6	1
34	R	47	R	EHL, 2 EDC injury, 2 Finger proximal phalanx fracture	Primary repair + K-wire fixation	94	10	3
35	L	55	R	Partial Achilles tendon injury	Primary repair	100	8	3
36	L	38	R	Partial patellar tendon injury	Primary repair	100	2	0
37	R	57	R	Partial patellar tendon injury	Primary repair	100	2	0
38	L	29	R	Gastrocnemius partial injury	Primary repair	94	5	3
39	L	41	R	Partial patellar tendon injury	Primary repair	93	6	2
40	L	53	R	Partial TAT injury	Primary repair	95	5	4
41	L	52	R	FHL, FDC injury	Primary repair	95	8	2

**Table 2:** Data of injury side and hand dominance

Hand dominance	Right lower extremity injury	Left lower extremity injury	P-value
Right-handed	7	30	0.012
Left-handed	2	2	
Total	9	32	

**Table 3:** Distribution of injured structures

Injured structures	Patient number (n)
Extensor hallucis longus (EHL) tendon	24
Proximal phalanx	8
Tibialis anterior tendon (TAT)	8
Extensor digitorum communis tendon	7

**Figure 2:**
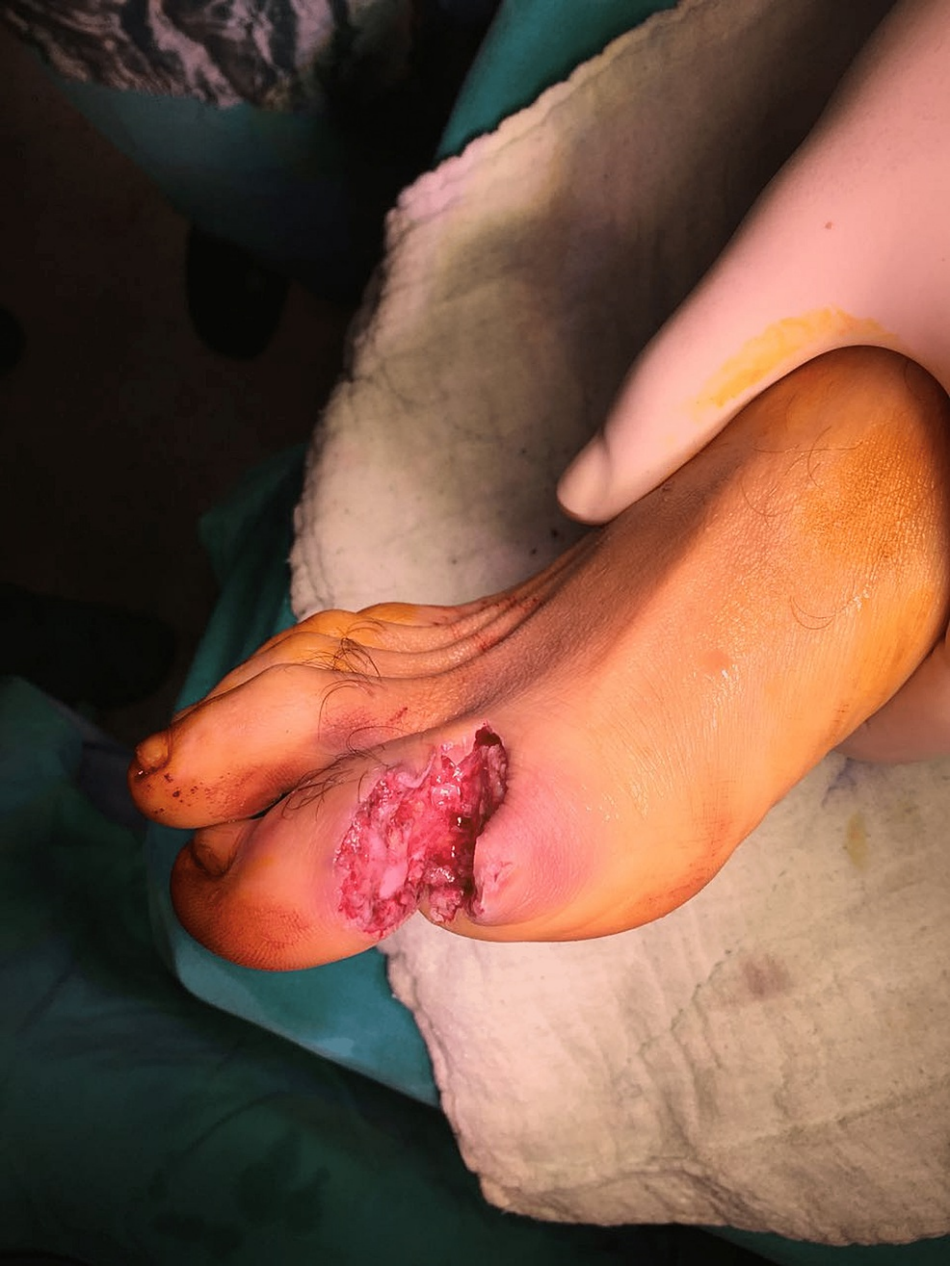
42-year-old male patient, right foot subtotal toe amputation

**Figure 3:**
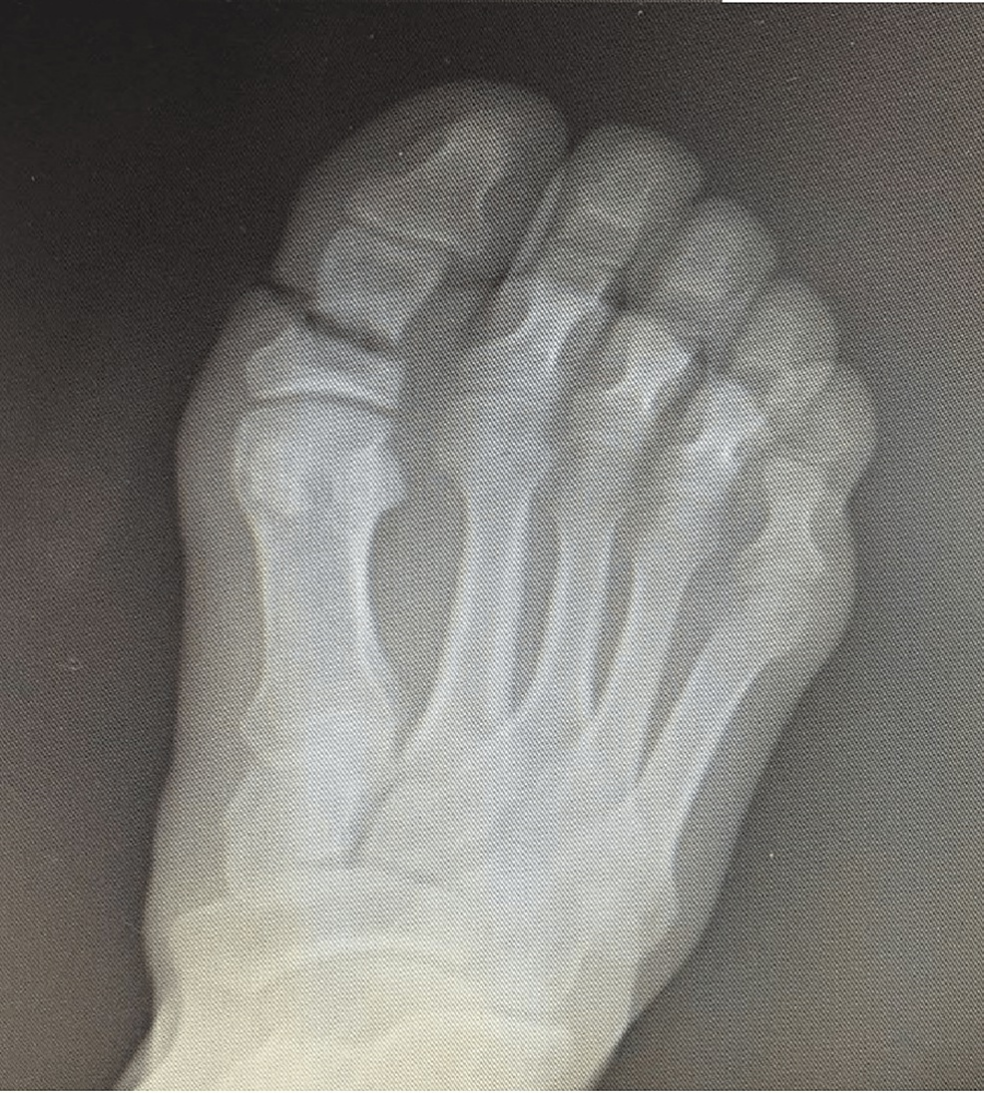
Subtotal toe amputation, AP radiograph

**Figure 4:**
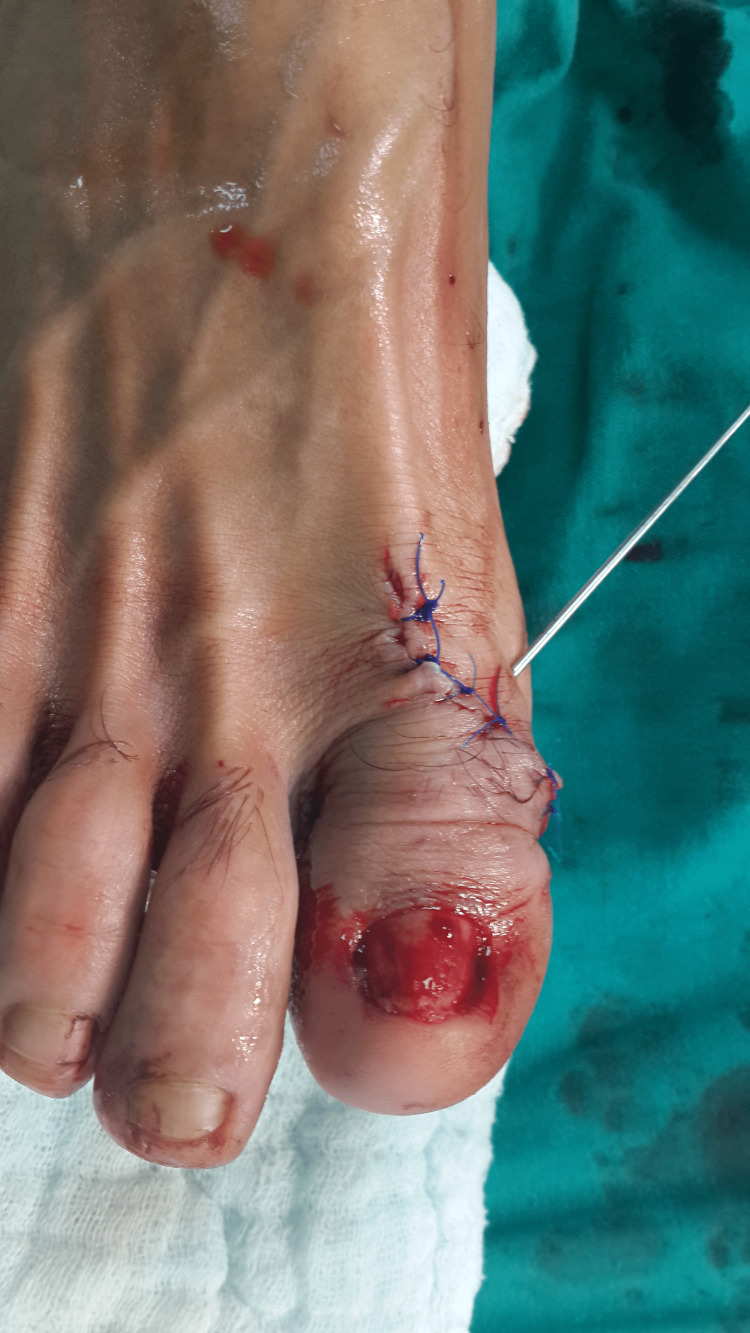
Intra-operative image of the patient

**Figure 5:**
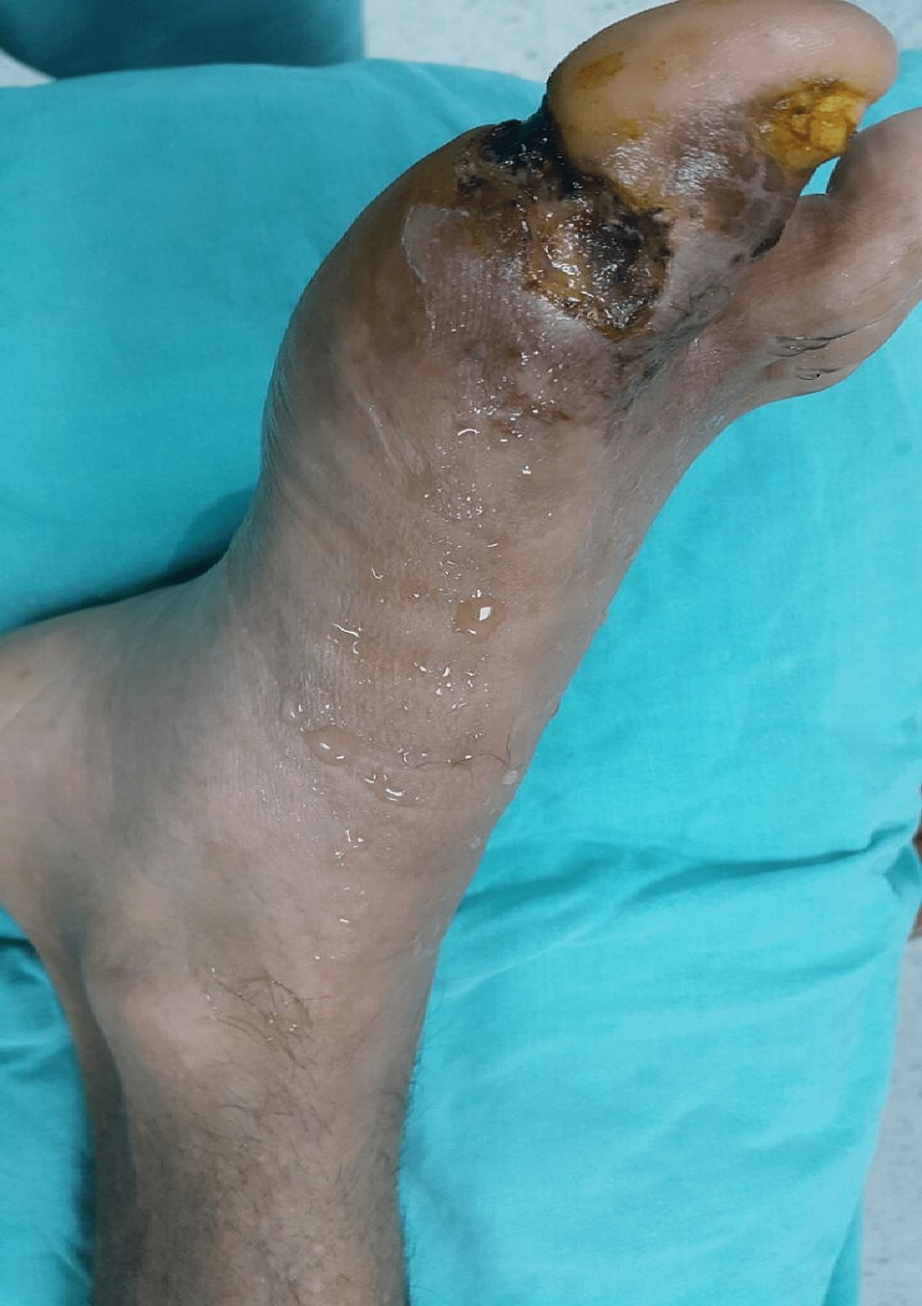
Post-operative: two months

Primary repair was performed for the related injuries in all cases. Except for one case, no wound problems, superficial or deep infection, nonunion, contracture, or similar complications were observed. First-generation cephalosporin (intravenous or oral) was given for five days to all patients who were operated or treated in the emergency department. In cases with fractures, metronidazole was given for five days, and aminoglycoside (gentamicin) was also given for three days. Except for one case, no secondary surgery was needed. In this case, who was treated for subtotal amputation of the toe, due to the development of circulation problems at the wound site in the third month, amputation was performed from the first metatarsophalangeal joint as the second surgery, and the stump was closed (Figure 2-5). The patient developed sudeck atrophy as a complication after the second surgery, which resolved in the sixth month. The mean AOFAS score of the patients was 97.4 ± 4.4 (74-100). The mean hospitalization length of stay was 2.95 ± 2.7 (0-15) days. The mean time to return to work was calculated as 6.17 ± 1.4 weeks (2-15).

## Discussion

Chainsaw injuries with associated morbidity are not uncommon in Turkey, a developing nation, although they are under-reported. During the fou-year period studied, there were 54 patients admitted to our hospital with a chainsaw injury. EHL tendon disruption was the most common injury in 24 (58.5%) of the 41 patients included in the study. Injuries from these woodworking tools often occur as 'do-it-yourself' home hobby work, such as maxillofacial or hand injuries [8,9]. In one study, lower extremity injuries were found to be 41.5% of chainsaw-related injuries [4]. In another study, injuries that cause loss of work occur mostly in the first four months of the year in the USA [10]. In our study, we saw that there were cases generally in the autumn and winter. Again, according to our study, there is an average of six weeks of job loss.

Although the mechanism of injury in foot injuries can vary widely, the chain machine creates a curvilinear and defective effect of the chain width in the tissues. It can also cause heat necrosis with the effect of the fast-rotating chain. We attribute our case resulting in amputation to this effect. Pieces of wood or working material may remain in damaged tissues. Debridement and wound cleaning are important to reduce the risk of contamination. The injury is primarily caused by the kickback from the surface that the tool cuts perpendicular to the axis. This results in the rotating chain coming directly to the operator. Facial injuries can occur if this kickback occurs when the operator is looking down along the axis of the cut [1-3]. We found that foot injuries are mostly the result of sagittal or parasagittal cuts and partial tissue loss.

When we categorize saw-related injuries by occupation, they tend to be older than occupationally injured patients [2,3]. 90.2% of our cases were not occupational professionals. In another study conducted in Turkey, a 26.5% fracture rate and 6.1% arterial injury were reported [7]. They stated that all of their cases were occupational injuries [7]. The absence of long bone fractures in our study can be attributed to this. We observed mostly phalanx fractures (eight patients) in 29.2% (12/41) patients, forefoot and midfoot fractures such as metatarsal (two patients), medial cuneiform (one patient), and navicular fractures (one patient). We attribute this to the fact that our patients see serious injuries that will cause job loss and that they use them in their own simple garden work.

Risk factors for chainsaw injuries are not different from those identified for general hand and face injuries. Although there were few occupational accident cases in our study, this tool is a high-powered machine designed for use by experienced people on the job. Therefore, fixed and temporary risk factors such as age, gender, experience, safety training (fixed risk factors), inappropriate tasks, work equipment, methods, and patient-related factors also apply to this device [8,11-13].

The chainsaw is a widely used tool in woodworking, both professionally and as a hobby. As these are preventable injuries, the use of protective equipment and trained persons can potentially result in a lower incidence [2,11]. A special lining must be used so that the toe of the shoe is made of steel and the front and sides of the feet have the greatest possible protection [11]. Some countries set standards for foot protection clothing with guidelines. Saw-protected trousers will greatly reduce the chance of operators' legs being lacerated. Thus, economic damage can be prevented due to the loss of work force caused by such injuries [12]. Good saw protection trousers, which will prevent accidents and injuries caused by the chainsaw's contact with the leg, are beneficial because they have the longest possible cutting time and the ability to block and compress the chain thanks to the synthetic fibers they contain [13]. This study may play a role in improving our knowledge of future chainsaw injuries and in the prevention and treatment of lower extremity injuries. A comprehensive understanding of the specific mechanisms of trauma and the resulting patterns of trauma is imperative for orthopedic surgeons and emergency professionals.

The limitation of our study is the small study group. We think that it will provide valuable information for studies with larger study groups.

## Conclusions

We observed that these accidents were preventable, and patients admitted to our hospital often had lower extremity injuries. These injuries are generally seen in men, in right-handed individuals with injuries to the left foot dorsum, and most commonly with EHL tendon injuries. Safety shoes should be worn as a precaution. A special lining must be used so that the toe of the shoe is made of steel and the front and sides of the feet have the greatest possible protection. Saw-protected trousers will greatly reduce the chance of operators' extremities being cut. This study improves our knowledge about chainsaw injuries and may play a role in the prevention and treatment of lower extremity injuries in the future.
